# *Bacillus velezensis* NDB mitigates *Aeromonas hydrophila*-induced enteritis in black sea bream (*Acanthopagrus schlegelii*) by enhancing intestinal immunity and modulating gut microbiota

**DOI:** 10.3389/fmicb.2025.1660494

**Published:** 2025-09-26

**Authors:** Zhixuan Zhang, Ze Wang, Zhonghua Wang, Haojia Yi, Xiurong Su, Rixin Wang, Tinghong Ming, Jiajie Xu

**Affiliations:** ^1^School of Marine Science, Ningbo University, Ningbo, Zhejiang, China; ^2^Microbial Development and Metabolic Engineering Laboratory, Ningbo University, Ningbo, Zhejiang, China; ^3^Ningbo Foreign Language School, Ningbo, Zhejiang, China; ^4^Shandong Beiyou Biotechnology Co., Ltd., Weifang, Shandong, China

**Keywords:** black sea bream, *Aeromonas hydrophila*, enteritis, *Bacillus velezensis*NDB, immune modulation, intestinal health

## Abstract

*Aeromonas hydrophila*-induced enteritis presents a significant challenge to the intensive aquaculture of black sea bream (*Acanthopagrus schlegelii*). Studies have shown that probiotic supplementation provides a promising alternative to conventional antibiotic applications for disease prevention in aquaculture. In this study, we investigated the protective effects of dietary supplementation with *Bacillus velezensis* NDB on the growth, immunity, and intestinal microbiota of black sea bream against *A. hydrophila*-induced intestinal damage. A total of one hundred and eight black sea bream fingerlings (initial body weight of 9.44 ± 0.03 g) were randomly assigned to three groups (total of nine tanks, twelve fish per tank): a control group (CON, normal diet), an infected group (AH, normal diet for 28 days followed by *A. hydrophila* infection at 1.0 × 10^7^ CFU/mL), and a probiotic-treated group (AH+NDB, diet supplemented with 1.43 × 10^8^ CFU/g *B. velezensis* NDB for 28 days, followed by *A. hydrophila* infection at 1.0 × 10^7^ CFU/mL). The results showed that the weight gain rate in the AH+NDB group was 139.44 ± 48.61%, which was significantly higher than that in the CN (126.81 ± 43.48%) and AH (132.48 ± 63.54%) groups. The pathological symptoms of black sea bream induced by *A. hydrophila*, including gill and abdominal hemorrhage, villus deformation, and inflammatory infiltration, were alleviated in the AH+NDB group. Histological and biochemical analyses showed the dietary supplementation of *B. velezensis* NDB enhanced antioxidant enzyme activities (SOD and CAT) and reduced lipid peroxidation (MDA) in the AH+NDB group. Compared to the AH group, the AH+NDB group exhibited significantly upregulated expression levels of anti-inflammatory markers (*il10* and *tgf-*β), and significantly downregulated levels of pro-inflammatory cytokines (*il1, tnf-*α, and *ifng*). Moreover, dietary supplementation with *B. velezensis* NDB increased the abundance of beneficial genera (e.g., *Bacillus* and *Ruegeria*), and decreased the abundance of opportunistic pathogenic genera (e.g., *Aeromonas* and *Vibrio*), thus enhancing the carbohydrate/amino acid biosynthesis and promoting the nucleoside and nucleotide biosynthesis to alleviate *A. hydrophila*-induced enteritis. Collectively, the study demonstrated that dietary supplementation with *B. velezensis* NDB can effectively promote growth performance and enhances immune function in black sea bream, thereby providing significant benefits for fish culture.

## 1 Introduction

Black sea bream (*Acanthopagrus schlegelii*) is widely distributed in the warm waters of the Northwest Pacific, particularly along the Chinese coastline. Its economic value and suitability for aquaculture have gained increasing attention in Southeast Asia (Ullah et al., 2022). The black sea bream is considered highly suitable for intensive farming and coastal breeding due to its hardy nature and relatively fast growth rate (Sagada et al., 2021). However, high-density habitats in aquaculture facilities increase physical proximity and fish stress, thereby facilitating the spread of bacterial diseases (Pan et al., 2023). Among these, bacterial enteritis is a leading cause of mortality in farmed fish species (Ofek et al., 2023). *Aeromonas hydrophila* is one of the most prevalent opportunistic pathogens in freshwater, marine, and estuarine environments, posing a significant threat to intensive aquaculture of marine fish (Abdella et al., 2024). The fish intestine serves as a primary interface between the host and its aquatic environment, harboring a diverse microbial community that plays a crucial role in host health (Li J. et al., 2019). Therefore, *A. hydrophila* infection can cause severe intestinal lesions and inflammation, leading to significant mortality and economic loss in aquaculture (Chen et al., 2024). Previous research has demonstrated that *A. hydrophila* mainly suppresses beneficial gut microbiota through direct toxin secretion, or indirectly induces microbial dysbiosis by triggering host immune responses, ultimately leading to intestinal damage and inflammation in fish (Liang et al., 2022). Notably, microbial ecological imbalance is closely related to the onset of enteritis, manifested as loss of appetite and slow growth, which may progress to fatal consequences in severe cases (Wang et al., 2023a). Furthermore, *A. hydrophila* infection has been demonstrated to significantly disrupt gut microbial communities, leading to an increased relative abundance of genera such as *Serratia, Candida arthromitus*, and *Faecalibacterium* (Pan et al., 2023). Recently, investigating and developing probiotic-based strategies to enhance immune function in black sea bream have received increasing attention.

Maintaining fish health serves as a fundamental prerequisite for ensuring sustainable development in both the fisheries and aquaculture industry. In recent years, numerous studies have shown that probiotic supplementation can provide promising alternatives to antibiotics in aquaculture and are crucial for the healthy development of fish production (Gao et al., 2024; Leong et al., 2023). Further researches indicate that these beneficial microorganisms in aquaculture animals not only improve their digestive enzyme activities, stimulate their host immune responses, and enhance their intestinal health, but also modulate their gut microbiota, thereby protecting them from pathogens (Kharwar et al., 2022; Xia et al., 2024). Among them, spore-forming *Bacillus* species have been widely recognized superior probiotic feed additives in aquaculture, owing to their numerous health benefits (Du et al., 2021; Liaqat et al., 2024). For example, dietary supplementation with *Bacillus* strains has been shown to improve disease resistance in red sea bream (Jang et al., 2023), and enhance growth performance and survival rates of (Herjayanto et al., 2021). *B. pumilus* has also been reported to reduce disease incidence in black sea bream by enhancing resistance to bacterial pathogens (Ramesh et al., 2015). Moreover, *B. velezensis* has been demonstrated to be one of the most widely recognized probiotic strains in aquaculture, primarily attributed to its potent antimicrobial characteristics (Chen et al., 2021; Li Z. et al., 2019). Li et al. (2023) reported that *B. velezensis* LB-Y-1 increased the abundance of beneficial bacterial genera, such as *Parasutterella* and *Rikenellaceae*, and decreased the abundance of pathogenic bacteria *Escherichia-Shigella*, indicating its potential application as a direct-fed microbial supplement or starter culture in fermentation. Furthermore, *B. velezensis* has demonstrated significant antimicrobial activity and growth-promoting effects across various aquaculture species, including hybrid grouper (*Epinephelus lanceolatus* ♂ × *E. fuscoguttatus*♀) (Li J. et al., 2019), golden carp (*Carassius auratus*) (Yi et al., 2018), Nile tilapia (*Oreochromis niloticus*) (Zhang et al., 2019) and *L. vannamei* (Wang et al., 2021). Currently, research on the probiotic effects of dietary *B. velezensis* supplementation on black sea bream remains relatively scarce.

**Figure 1:**
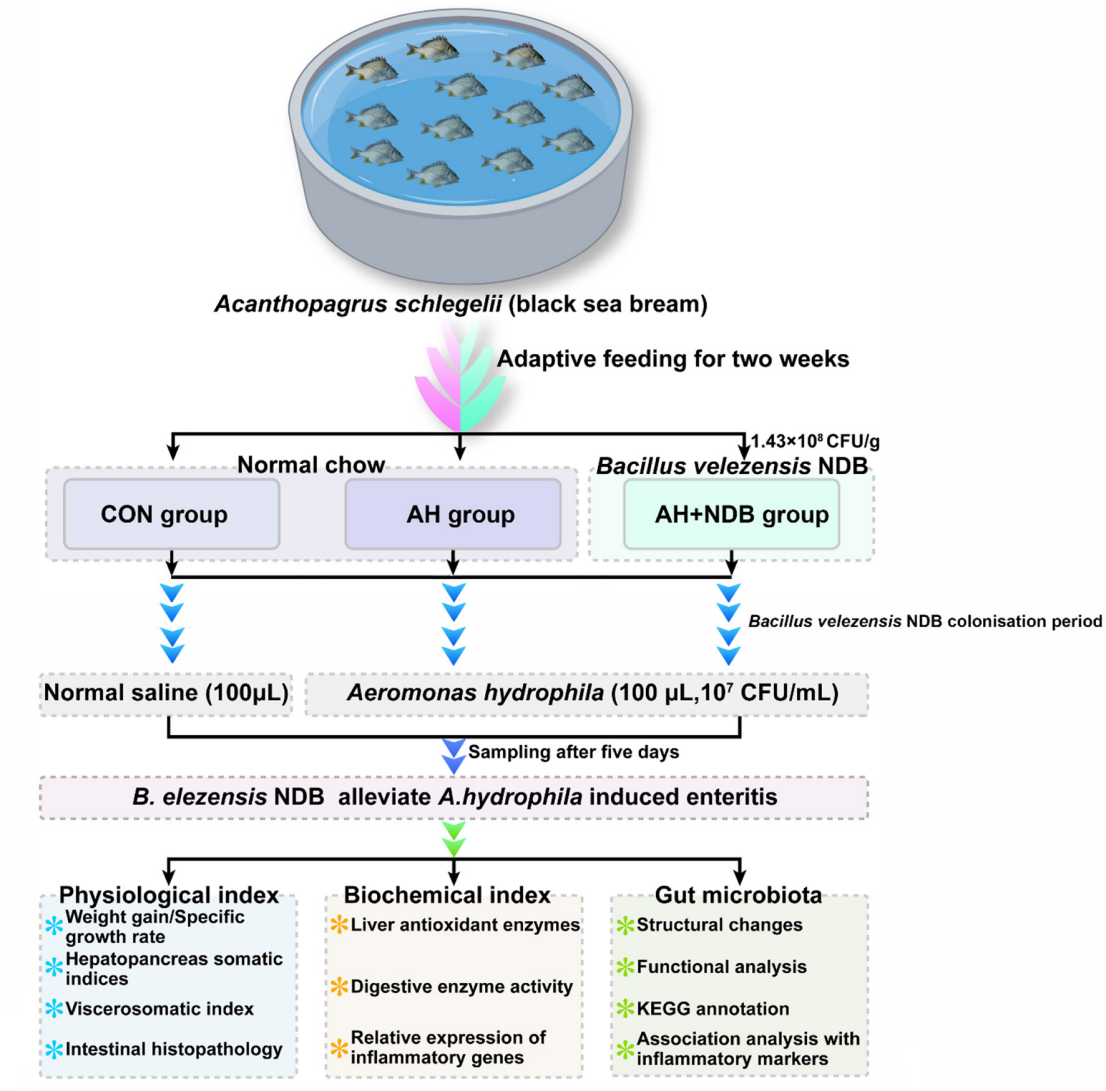
Experimental design of feeding and infection trials with black sea bream (*Acanthopagrus schlegelii*). After acclimation, black sea bream were randomly divided into three groups *(n* = 12). Fish in the CON group were fed the control diet, while those in the AH+NDB group were fed the *B. velezensis* NDB-supplemented diet (1.43 × 10^8^ CFU/g) for 28 days. On day 28, fish in the AH and AH+NDB groups were intrarectally injected with 100 μL of *A. hydrophila* (1 × 10^7^ CFU/mL), while the CON group received 100 μL of sterile saline. Samples were collected five days post-challenge for physiological and histological analysis. Note: During the two-week acclimation, all fish were fed with the commercial control diet.

Our previous study demonstrated that *B. velezensis* NDB, a probiotic strain isolated and characterized from Xiangshan Harbor seawater, suggested antimicrobial activity against 12 pathogenic bacteria (including *A. hydrophila*) as revealed by whole genome sequencing analysis (Wang Z. et al., 2024). In the present study, we explored the effects of dietary supplementation with *B. velezensis* NDB on growth performance, intestinal mucosal integrity, inflammatory gene expression, and gut microbiota composition in black sea bream challenged with *A. hydrophila* (Figure 1). This study potentially provides a valuable insight into the application of *B. velezensis* NDB as a functional feed additive in black sea bream aquaculture.

## 2 Materials and methods

### 2.1 Preparation of bacterial strain

A probiotic strain, *Bacillus velezensis* NDB, previously isolated from seawater in Xiangshan Harbor (Wang Z. et al., 2024). The strain was cultured on Luria–Bertani (LB) agar plates at 37 °C for 24 h. Single colonies were subsequently inoculated into LB broth and incubated at 37 °C for 14 h. *A. hydrophila* was cultured at 28 °C for 14 h and harvested by centrifugation at 5000 rpm for 10 min. The bacterial pellets were resuspended in phosphate-buffered saline (PBS) and serially diluted to achieve final concentrations of 1.43 × 10^8^ CFU/g for *B. velezensis* NDB and 1.0 × 10^7^ CFU/mL for *A. hydrophila*. The probiotic suspension is uniformly sprayed onto the surface of commercial feed pellets at a ratio of 10% (v/w), then air-dried at 30 °C for 2 h. Once the feed surface is dry, it can be fed, ensuring that the probiotics are evenly attached to the feed.

### 2.2 Fish preparation and aquacultural experiment

A total of 108 black sea bream fingerlings (9.44 ± 0.03 g body weight) were obtained from Xiangshan Port Aquaculture Co., Ltd., and reared at the Ningbo University Aquaculture Pilot Base. For acclimatization, the experimental fish were randomly allocated in nine 100 L fiber-reinforced tanks *(n* = 12 fish/tank) in a closed water system at 24.5 °C with continued aeration, 12:12 h photoperiod, and water pH = 8 for two weeks. Fish were fed daily with a commercial diet (Zhejiang Qiangpu Biotechnology Co., LTD.) of 3 mm diameter containing contains 45.0% crude protein and 10.5% crude fat. Once the two-week acclimation period elapsed, each tank containing 12 fish was randomly assigned into one of the three experimental groups: the control (CON) group, the black sea bream challenged with *A. hydrophila* (AH) group, and the dietary supplementation of *B. velezensis* NDB for black sea bream infected by *A. hydrophila* (AH + NDB) group. Black sea bream were obtained from Xiangshan Harbor Aquatic Species Co., LTD., and the feed was procured from Zhejiang Qiangpu Biotechnology Co., LTD. acclimation at the Ningbo University aquaculture facility, fish were randomly allocated into three groups (*n* = 12 per group): control (CON) group, the black sea bream challenged with *A. hydrophila*-challenged (AH), and *A. hydrophila*-challenged with dietary *B. velezensis* NDB supplementation (AH + NDB). Fish in the CON and AH groups were fed normal diets, while fish in the AH + NDB group were fed 1.43 × 10^8^ CFU/g of *B. velezensis* NDB (Monzón-Atienza et al., 2022), administered manually twice daily (09:00 and 17:00). The feeding rate was initially set at 2% of body weight and adjusted daily based on intake. Throughout the 4-week experimental period, the seawater temperature (24.5 ± 0.2 °C) and dissolved oxygen (9.6 ± 0.3 mg/L) were maintained using automated monitoring systems. Water quality parameters were rigorously controlled as follows: pH 7.8–8.2 (daily measurement with HANNA HI98107 pen-type pH meter), salinity 30–32 ppt (daily monitoring using ATAGO handheld refractometer), total ammonia nitrogen (TAN) < 0.05 mg/L (measured twice weekly using HACH ammonia test kit), and nitrite nitrogen (${\text{NO}}_{2}^{-}$-N) < 0.02 mg/L (measured twice weekly using the HACH nitrite test kit). All parameters are maintained within the optimal range for black sea bream growth.

### 2.3 Assessment of growth parameters of black sea bream

After 28 days of feeding, the total number and weight of black sea bream in each fish tank were recorded to calculate survival rate, Weight Gain Rate (WGR), Specific Growth Rate (SGR), Hepatosomatic Index (HSI), Viscerosomatic Index (VSI), and Condition Factor (CF), using the formulas described previously (Saravanan et al., 2021):


$$\begin{array}{l} \text{WGR}=\left(\text{W}-{\text{W}}_{0}\right)/W_{0}\times \;100\%\\ \text{SGR}=\left(\text{lnW}-\text{ln}{\text{W}}_{0}\right)/t\times \;100\%\\ \text{HSI}={\text{W}}_{\text{h}}/W\times \;100\%\\ \text{VSI}={\text{W}}_{\text{v}}/W\times \;100\%\\ \text{CF}=\text{W}/{\text{L}}^{3}\times l00\%\\ \text{SR}={\text{N}}_{\text{t}}/N\times l00\% \end{array}$$

Note: W_0_ = initial average weight of black sea bream; W = final average weight of black sea bream; N = initial number of black sea bream; N_t_ = final number of black sea bream; t (d) = test days; L (cm) = body length of each fish; W_h_ (g) = liver weight of each fish; W_v_ (g) = visceral weight of each fish.

### 2.4 Pathogen challenge test and sample collection

One day before the bacterial challenge, thirty six fish were placed in 100 L aerated plastic tanks and fasted to reduce intestinal fecal content. Fish were anesthetized by bath immersion with 60 mg/L MS-222 (Sigma, E10521-10G) for 5 min before challenge. The fish were immersed in the anesthetic solution until they lost balance (approximately 3-5 min), and rectal injection was performed by inserting the needle approximately 3 cm into the anus. Preliminary experiments determined that an *A. hydrophila* concentration of 1 × 10^7^ CFU/mL would cause approximately 50% mortality (Pan et al., 2023). Fish in the AH and AH+NDB groups received 100 μL of the bacterial suspension, while the CON group was administered 100 μL of sterile saline. After injection, fish were held inverted for 2–3 min to prevent leakage.

At 5 days post-infection, six fish were randomly sampled from each group (for serum, tissue, molecular, and microbiological analyses). Blood samples were collected from the caudal vein and centrifuged at 3000 rpm for 15 min at room temperature. Serum was stored at −80 °C for further analysis (Kong et al., 2017). Assay kits for Superoxide Dismutase (SOD), catalase (CAT), malondialdehyde (MDA), and gastric protease were obtained from Jiancheng Bioengineering Institute (Nanjing, China). TransZol Up, TransScript All-in-One First Strand cDNA Synthesis Kit, DEPC-treated water, SuperMix for qPCR (One Step gDNA Removal), and TransStart Green qPCR SuperMix were purchased from Beijing Quanshijin Biotechnology Co., Ltd.

### 2.5 Histological analysis

Intestinal tissues exhibiting significant pathological lesions were retrieved from−80 °C storage and fixed in 4% paraformaldehyde. Samples were then embedded in paraffin, sectioned at 4 μm thickness, dewaxed, and stained with Hematoxylin and Eosin (HE). The sections were examined and photographed using an Olympus BX51 light microscope at × 100 and × 400 magnification. Tissue damage was independently assessed via double-blind scoring by two experienced pathologists. The severity of colonic histological injury was assessed using a scoring system, which was quantitatively evaluated three parameters: inflammation severity (graded 0–3 with 3 as the maximum score), crypt damage (graded 0–5 with 5 as the maximum score), and ulceration extent (graded 0–3 with 3 as the maximum score) as previously described (Wang et al., 2022). All experiments were performed with six replicates.

### 2.6 Liver sample for immunological measurement

Liver tissue samples were prepared as a 10% homogenate, and the supernatant was collected by centrifugation at 4000 rpm for 10 min at 4 °C. The supernatant was aliquoted into centrifuge tubes for subsequent measurements. The activities of SOD, CAT, MDA, and gastric protease were analyzed. These biochemical indices were measured using a commercial kit from Jiancheng Bioengineering Institute of Nanjing.

### 2.7 RNA isolation and quantitative real-time PCR (qRT-PCR) analysis

Total RNA was extracted from black seabream intestinal tissue by the TransZol Up reagent kit. The quality and quantity of RNA were determined by agarose gel electrophoresis and NanoDrop 2000 spectrophotometry (Vastech Inc., China). Prior to cDNA synthesis, genomic DNA was removed from total RNA using the FastQuant RT Kit with gDNase (Takara), followed by synthesis of complementary DNA (cDNA). Take 1 μg of total RNA, add 4 μL of 5 × TransScript All-in One SuperMix for qPCR and 1 μL of gDNA Remover, and dilute with RNase-free water to a total volume of 20 μL. Mix well, incubate at 42 °C for 15 min, and finally inactivate the TransScript RT/RI and DNA Remover reagents by heating at 85 °C for 5 s. The synthesized cDNA is diluted to 30 ng/μL and can be used as an RT-PCR template. The Rotor-Gene 6000 is used to amplify the target gene and internal control gene from the cDNA, and a standard curve is created using β*-actin* as the internal control gene to ensure that the target gene and internal control gene have similar amplification efficiencies. After validating the standard curve, the reaction system consists of 2 μL cDNA template, 0.8 μL forward primer (10 μM), 0.8 μL reverse primer (10 μM), 10 μL SYBR Premix Ex TaqTM III, and ddH_2_O to a total volume of 20 μL. The reaction programme was as follows: 95 °C pre-denaturation for 10 min, 95 °C denaturation for 10 s, 55 °C annealing for 10 s, 72 °C extension for 20 s, 40 cycles, and a final extension for 10 min. Each plate included a No-Template Control (NTC) to rule out reagent contamination. The Ct values were automatically calculated by the detector using the standard curve, and relative quantitative analysis was performed using the 2^−Δ*ΔCt*^ method. PCR primers were designed using NCBI Primer BLAST based on the NCBI database and synthesized by Shanghai Sangon Biotechnology Co., Ltd. Primer sequences are summarized in Table 1.

**Table 1:** Sequences of the primers used for qPCR analysis.

**Item**	**Forward primer (5^′^to 3^′^)**	**Reverse primer (5^′^to 3^′^)**
*tnf-α*	GACACCTCACACCTCTCAGCC	GCAAACACACCGAAGAAGGTC
*il1*	AGAATCAAGGAGGGAGACAGGA	GTAGAGGAAGACAGAGACCAA
*il8*	CCGCTGCATCCAAACAGAGAG	ATCACTTTCTTCACCCAGGGAGC
*il10*	CCGAGACTTCTACGAAGCAAAC	CTGGATGGACTGCATGTGAGG
*ifng*	CATGGGTGGCATTTTGGACA	CAGCTCCTGGACCTTCTTCA
*myd88*	AGCCGTACCCAGAACCAG	CGGAGCACGAAGTAAACG
*IκB-α*	GTGAGGTGGAAGGGAGTG	AACAGCGTAATGGTCGTG
*tgf-β*	TGTCTCCCCTACCCGCCGTCATC	ACCTCGCCTCCCGCTTCATCACT
*claudin7*	ACTGTTGGGGTTTTTCCTGTCTC	GTGATGATGTTGTCCCCGATGTA
*nfkb*	TGAATTACCCCAACAGCATCG	TTGGGTGTCCTGACACAAACC
*β-actin*	ACAGTGCCCATCTATGAAGGCT	GGCTGTGGTGGTGAAGGAGTAG

β*-actin*, reference gene; *tnf-*α, tumor necrosis factor alpha; *il1*, interleukin 1; *il8*, interleukin 8; *il10*, interleukin 10; *ifng*, interferon gamma; *myd88*, MYD88 innate immune signal transduction adaptor; *I*κ*B-*α, inhibitor of NF-κB alpha; *tgf-*β, transforming growth factor beta; *claudin7*, Claudin-7 (tight junction protein); *nfkb*, nuclear factor kappa β.

### 2.8 Intestinal DNA extraction and 16S rRNA gene sequencing

The PowerFecal^®^ DNA Isolation Kit (MoBio Laboratories, Carlsbad, CA, United States) was employed to extract DNA from intestinal samples, in accordance with the manufacturer's instructions, with six samples selected from each group. Extraction negative controls were included to monitor for potential contamination. The quantities and qualities of the extracted DNA were verified through a NanoDrop 2000 (ThermoFisher, Wilmington, DE, United States) and gel electrophoresis. The V3–V4 region of bacterial 16S rRNA gene was amplified by PCR using the primers of ~20 ng with the following primer order: forward primers: 5′-ACTCCTACGGGAGGCAGCA-3′; reverse primers: 5′-GGACTACHVGGGTWTCTAAT-3′ (Pan et al., 2021). The PCR negative controls (no-template controls) were also included in the amplification step. The high-throughput sequencing was performed by the Shanghai Personal Biotechnology Co., Ltd. (Shanghai, China) on an Illumina platform (Illumina, San Diego, CA, US) with a PE250 strategy in accordance with the previously described method (Wang et al., 2020). After quality filtering and denoising using the DADA2 plugin in QIIME2 (version 2022.11), an average of 50,000 high-quality reads per sample were retained for downstream analysis. To minimize batch effects, samples were randomized across sequencing lanes during library preparation and sequencing.

### 2.9 Bioinformatics and statistical analysis

Microbiome bioinformatics analysis was conducted using QIIME2 (version 2022.11), following the official tutorials. Before analysis, the raw sequence data underwent demultiplexing, quality filtering, denoising, merging, and chimera removal using the DADA2 plugin to generate Amplicon Sequence Variants (ASVs). Taxonomic classification of ASVs was performed using the SILVA 138 database. Prior to downstream alpha and beta diversity analyses, all samples were rarefied to an even depth of 30,000 sequences per sample to account for uneven sequencing effort. Mothur version 1.36.0 was employed to analyze alpha diversity, utilizing the observed species index, Chao1 index, Shannon index, and Simpson index. Principal Coordinate Analysis (PCoA) based on weighted UniFrac distances was executed to assess beta diversity. Finally, the RDP Classifier software was utilized to annotate species and analyze community changes in the processed sequences (Gao et al., 2022). Linear discriminant analysis effect size (LEfSe) was analyzed using the R statistical package (v3.1.1). Spearman correlation analysis was conducted using the R psych and pheatmap packages (v3.1.1). Functional prediction of the gut microbiota for Kyoto Encyclopedia of Genes and Genomes (KEGG) and Metabolic Pathways From all Domains of Life (MetaCyc) pathways was performed using the Phylogenetic Investigation of Communities by Reconstruction of Unobserved States (PICRUSt2) software.

### 2.10 Statistical analysis

All experimental data are presented as mean ± standard deviation (SD). The normality of data distribution was assessed using the Shapiro-Wilk test, and the homogeneity of variances was verified using Levene's test. Data conforming to normal distribution were analyzed by one-way analysis of variance (ANOVA) followed by Tukey's *post hoc* test, while the data that did not conform to a normal distribution were analyzed using the Mann-Whitney test. All statistical analyses were performed using SPSS software (version 26.0) and GraphPad Prism (version 9.0). A value of *p* < 0.05 indicates statistically significant differences.

## 3 Results

### 3.1 Growth performance and physiological indicators of black sea bream

**Figure 2:**
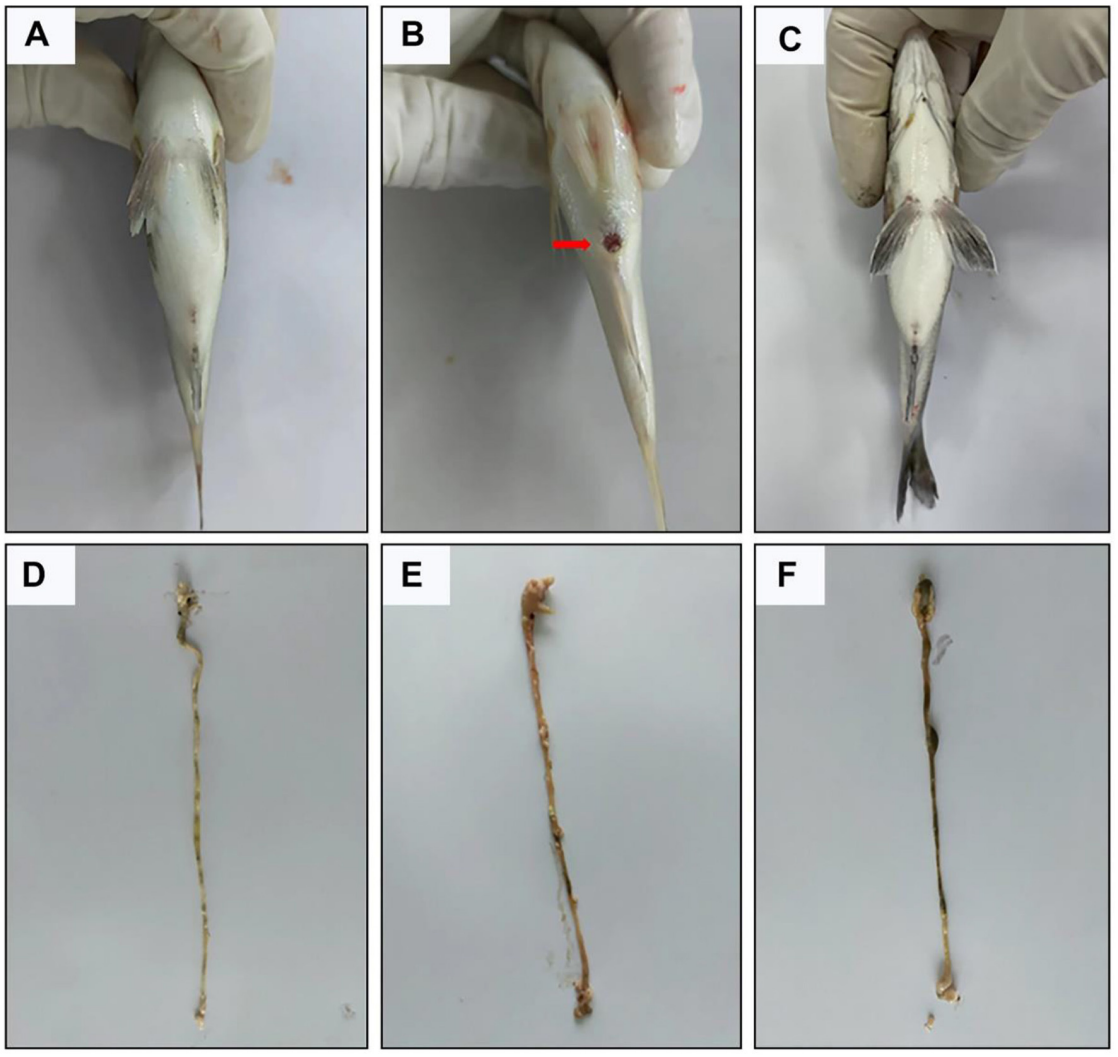
Representative pictures of the effect of *B. velezensis* NDB on intestinal lesions in black sea bream. **(A–C)** Perianal inflammatory symptoms in the CON, AH and AH+NDB groups. **(D–F)** Intestinal status in the CON, AH and AH+NDB groups.

Injecting 100 μL of 1 × 10^7^ CFU/mL of *A. hydrophila* solution or an equivalent volume of physiological saline was administered via anal injection into black sea bream. Mild inflammation in the anal region was observed in both the CON and AH+NDB groups on day one post-injection (Figure 2A), which largely subsided by the second day. In contrast, fish in the AH group exhibited marked symptoms including lethargy, reduced swimming activity, and petechial hemorrhages at the fin bases. Additionally, hemorrhagic lesions were evident in the gills, skin, and abdominal areas, accompanied by pronounced abdominal distension (Figure 2B). Upon dissection, ascitic fluid accumulation and varying degrees of hemorrhage in the skin and intestines were noted, with histological signs of tissue deformation and necrosis (Figure 2E). The intestinal morphology in the CON and AH+NDB groups, by contrast, showed no significant lesions (Figures 2D, F). Cumulative mortality rates at the end of the challenge period (5 days) were 0% in the CON group (0 of 36 fish), 18.52% in the AH group (2 of 36 fish), and 5.56% in the AH+NDB group (1 of 36 fish). Mortality commenced on day two post-infection, reaching 18.52%, indicative of typical *A. hydrophila*-induced pathology. In contrast, the AH+NDB group exhibited only mild anal inflammation, with no significant lesions observed elsewhere (Figure 2C). Growth performance parameters of black sea bream are summarized in Table 2. Unexpectedly, no significant differences were detected among groups in terms of WGR, SGR, VSI, HSI, CF and SR (*p* > 0.05).

**Table 2:** Physiological parameters of black sea bream in the CON, AH and AH + NDB groups.

**Item**	**CON**	**AH**	**AH + NDB**	***p*-value**
WGR (%)	126.81 ± 43.48	132.48 ± 63.54	139.44 ± 48.61	0.891
SGR (%)	3.20 ± 0.54	3.14 ± 1.49	3.23 ± 1.01	0.983
VSI (%)	10.71 ± 2.11	10.70 ± 2.60	10.91 ± 1.31	0.972
HIS (%)	3.18 ± 0.60	3.21 ± 1.16	3.13 ± 0.57	0.969
CF (%)	1.53 ± 0.33	1.57 ± 0.12	1.69 ± 0.17	0.421
SR (%)	97.22 ± 4.81	94.44 ± 4.81	97.22 ± 4.81	0.654

CON, control group; AH, the black sea bream challenged with *A. hydrophila* group; AH+NDB, *A. hydrophila*-challenged with dietary *B. velezensis* NDB supplementation group. WGR, weight gain rate; SGR, specific growth rate; VSI, viscerosomatic index; HSI, hepatosomatic index; CF, condition factor; SR, survival rate. Values are presented as mean ± SD (*n* = 12). The *p*-value was obtained by one-way ANOVA. No significant differences were found among groups for any parameters (*p* > 0.05).

### 3.2 Pathological changes in intestinal tissue

**Figure 3:**
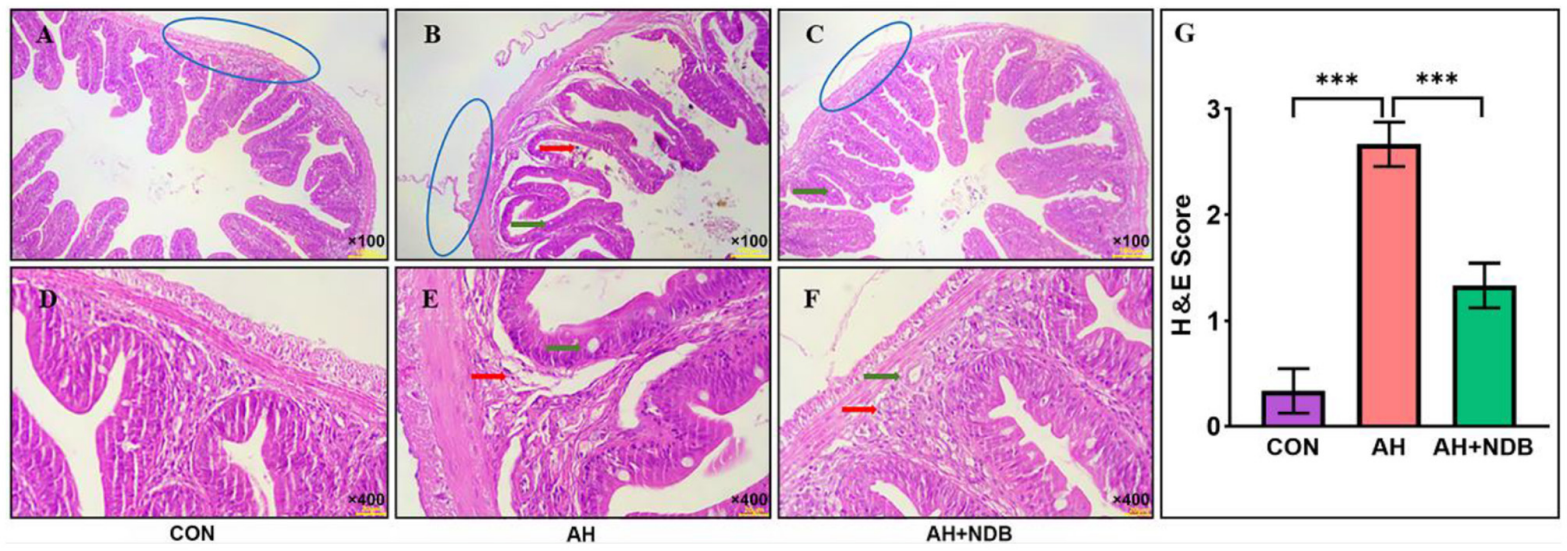
Photomicrographs of hematoxylin-eosin (HE) staining cross sections of the effect of *B. velezensis* NDB on histomorphological changes of intestinal segments of fish infected with *A. hydrophila*. Pathological changes of the intestine in the **(A–D)** CON, **(B–E)** AH, and **(C–F)** AH+NDB groups. Histological analysis of fish intestine sections under different conditions using HE staining. Blue circles: mucosal layer, red circles: cup cells, green arrows: inflammatory cells. **(G)** Histopathological score. Histopathological lesions in the intestine were scored on a scale of 0-3: 0 = no lesions; 1 = mild lesions affecting <25% of the tissue; 2 = moderate lesions affecting 25-50%; 3 = severe lesions affecting >50% of the tissue. The data are expressed as the means ± SD (*n* = 6). The symbol “*” indicates any group compared with the AH group. ***p < 0.001 by one-way ANOVA and Tukey's *post-hoc* test.

Histopathological analysis was was conducted to assess intestinal tissue damage induced by *A. hydrophila* infection. In the CON group, no macroscopic signs of ulceration or congestion were observed, and HE-stained sections showed normal intestinal architecture with intact epithelium (Figures 3A–D). In contrast, the AH group exhibited notable pathological changes, including congestion, epithelial erosion, villus deformation with widened interspaces, epithelial rupture and detachment, reduced goblet cell numbers, and marked infiltration of inflammatory cells (Figures 3B–E). In the AH+NDB group, intestinal morphology was largely preserved, and characterized by intact epithelial lining, abundant goblet cells, and minimal inflammatory cell infiltration (Figures 3C–F). As shown in Figure 3G, dietary supplementation with *B. velezensis* NDB significantly decreased histopathological score as compared to the AH group (*p* < 0.05).

### 3.3 Changes in digestive enzyme activities and liver antioxidant capacity

**Figure 4:**
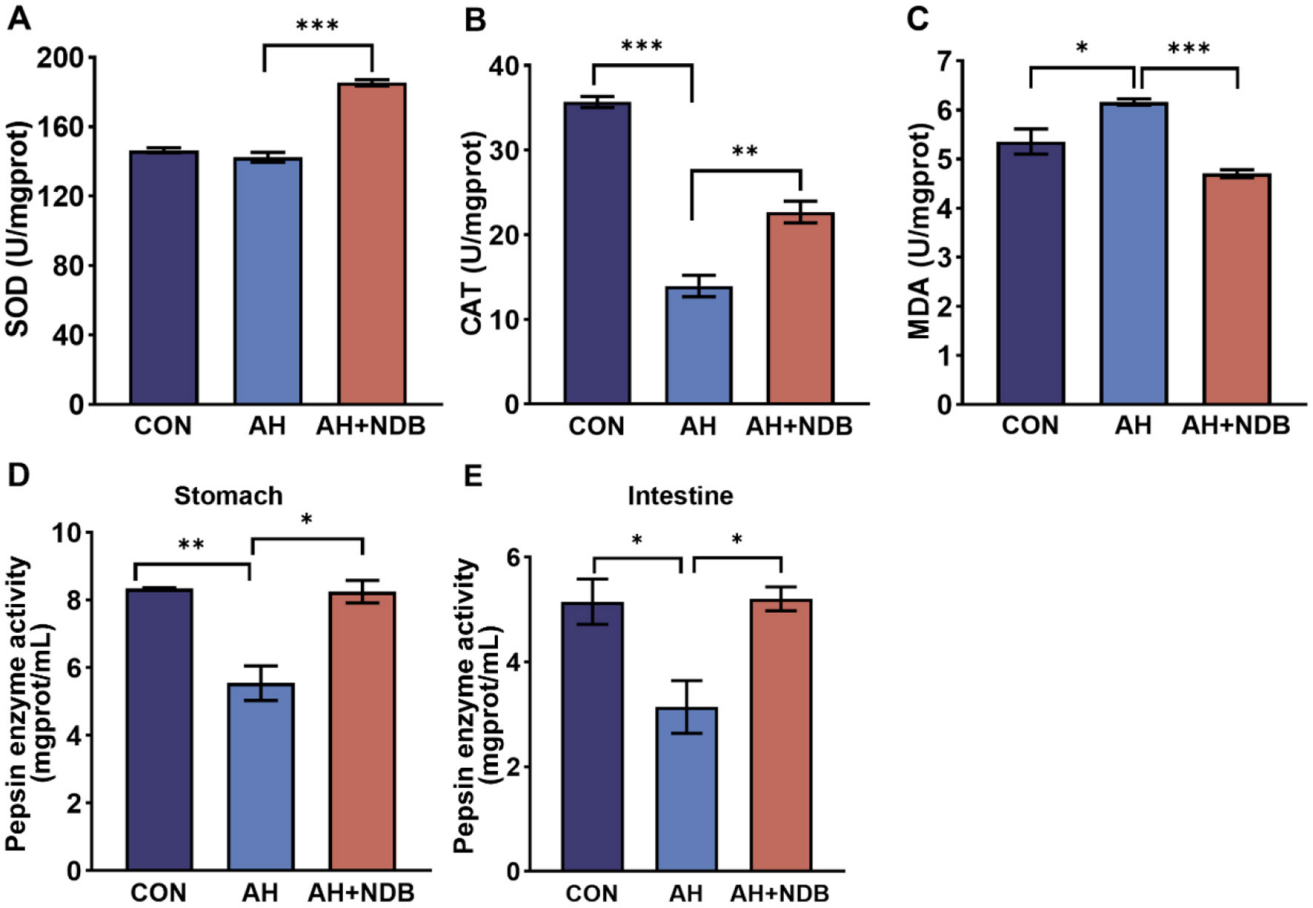
Effects of *B. velezensis* NDB on liver antioxidant enzyme activity and digestive enzyme function. **(A)** Superoxide dismutase (SOD) activity in the liver. **(B)** Catalase (CAT) activity in the liver. **(C)** Malondialdehyde (MDA) levels in the liver. **(D)** Pepsin enzyme activity in the stomach. **(E)** Pepsin enzyme activity in the intestine. The data are expressed as the mean ± SD (*n* = 12). * *p* < 0.05, **p < 0.01 and ***p < 0.001 by one-way ANOVA and Tukey's *post-hoc* test.

Dietary supplementation with *B. velezensis* NDB significantly increased superoxide dismutase (SOD) activity (185.39 ± 3.52 U/mgprot) (Figure 4A) and catalase (CAT) activity (22.70 ± 2.21 U/mgprot) (*p* < 0.01) in the livers compared to that of the AH group (Figure 4B). Compared with the CON group, the activity of CAT in the liver of black sea bream infected with *A. hydrophila* in the AH group was significantly decreased, and the content of Malondialdehyde (MDA) was significantly increased (*p* < 0.05) (Figure 4C). However, the MDA content of the AH+NDB group was significantly lower than that of the AH group (4.71 ± 0.14 U/mgprot) (*p* < 0.001). Compared with the CON group, *A. hydrophila* infection significantly decreased gastric pepsin activity (5.540 ± 0.001 mgprot/mL) and intestinal pepsin activity (3.14 ± 0.75 mgprot/mL) (*p* < 0.05) in the AH group (Figures 4D, E). Compared with the AH group, dietary supplementation with *B. velezensis* NDB significantly increased gastric pepsin activity (8.24 ± 0.34 mgprot/mL) and intestinal gastric pepsin activity (5.2 ± 2.0 mgprot/mL) (*p* < 0.05), the values of which nearly approach those of the CON group.

### 3.4 Changes in inflammatory pathway-related genes

**Figure 5:**
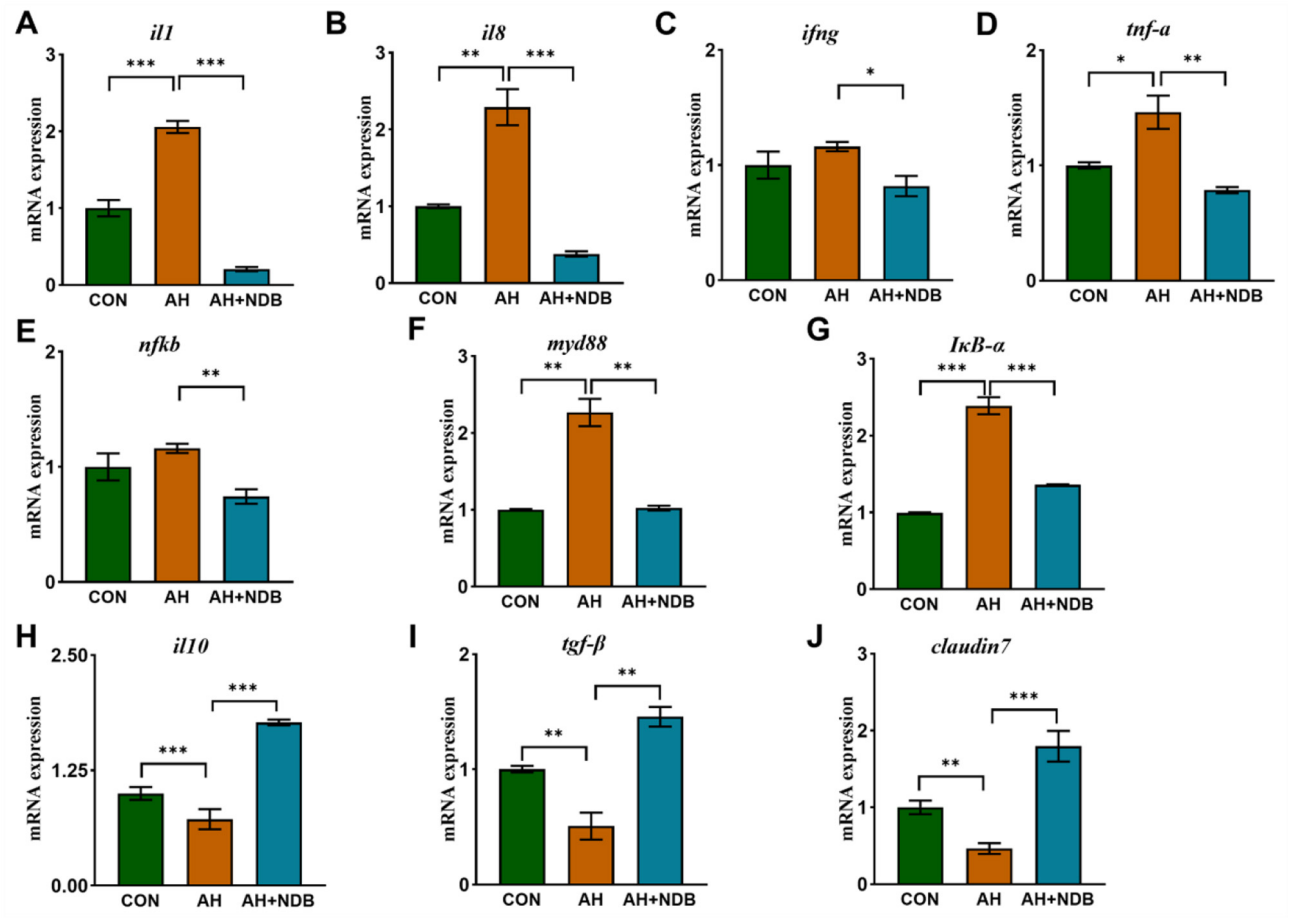
The effect of *B. velezensis* NDB on gene expression of inflammatory and anti-inflammatory markers in the intestine. **(A–G)** The mRNA expression levels of intestinal inflammatory genes (*il1, il8, ifng, tnf-*α, *nf*κ*b, myd88*, and *I*κ*B-*α) was detected by RT-PCR. **(H–J)** The mRNA expression levels of the anti-inflammatory factors-related genes (*il10* and *tgf-*β) and the tight junction gene *claudin7*. The data are expressed as the mean ± SD (*n* = 12). * *p* < 0.05, **p < 0.01 and ***p < 0.001 by one-way ANOVA and Tukey's *post-hoc* test.

As shown in Figures 5A–D, the intestinal mRNA expression levels of *il1, il8, ifng* and *tnf-a* in the AH group were higher than those of the CON group (*p* < 0.05). However, dietary supplementation with *B. velezensis* NDB in the AH+NDB group markedly downregulated the expression of pro-inflammatory genes, including *il1, il8, ifng* and *tnf-a* as compared to the AH group (*p* < 0.05). Furthermore, the gene expression levels associated with the NF-κB signaling pathway (*nfkb, myd88* and *I*κ*B-*α) were examined as depicted in Figures 5E–G. The AH group exhibited significantly increased expression levels of *nfkb, myd88* and *I*κ*B-*α compared to the CON group (*p* < 0.01), whereas the levels of which were significantly decreased in the AH+NDB group (*p* < 0.01). In contrast, dietary supplementation with *B. velezensis* NDB in the AH+NDB group markedly upregulated the expression of the anti-inflammatory genes (*il10* and *tgf-*β) and the tight junction gene *claudin7* as compared to the AH group (Figures 5H–J). Compared with the CON group, the gene expression levels of *il10, tgf-*β and *claudin7* were decreased in the AH group (*p* < 0.01), whereas the levels of which were significantly increased in the AH + NDB group (*p* < 0.01).

### 3.5 Changes in intestinal microbial composition

#### 3.5.1 Richness and diversity of gut microbiota

**Figure 6:**
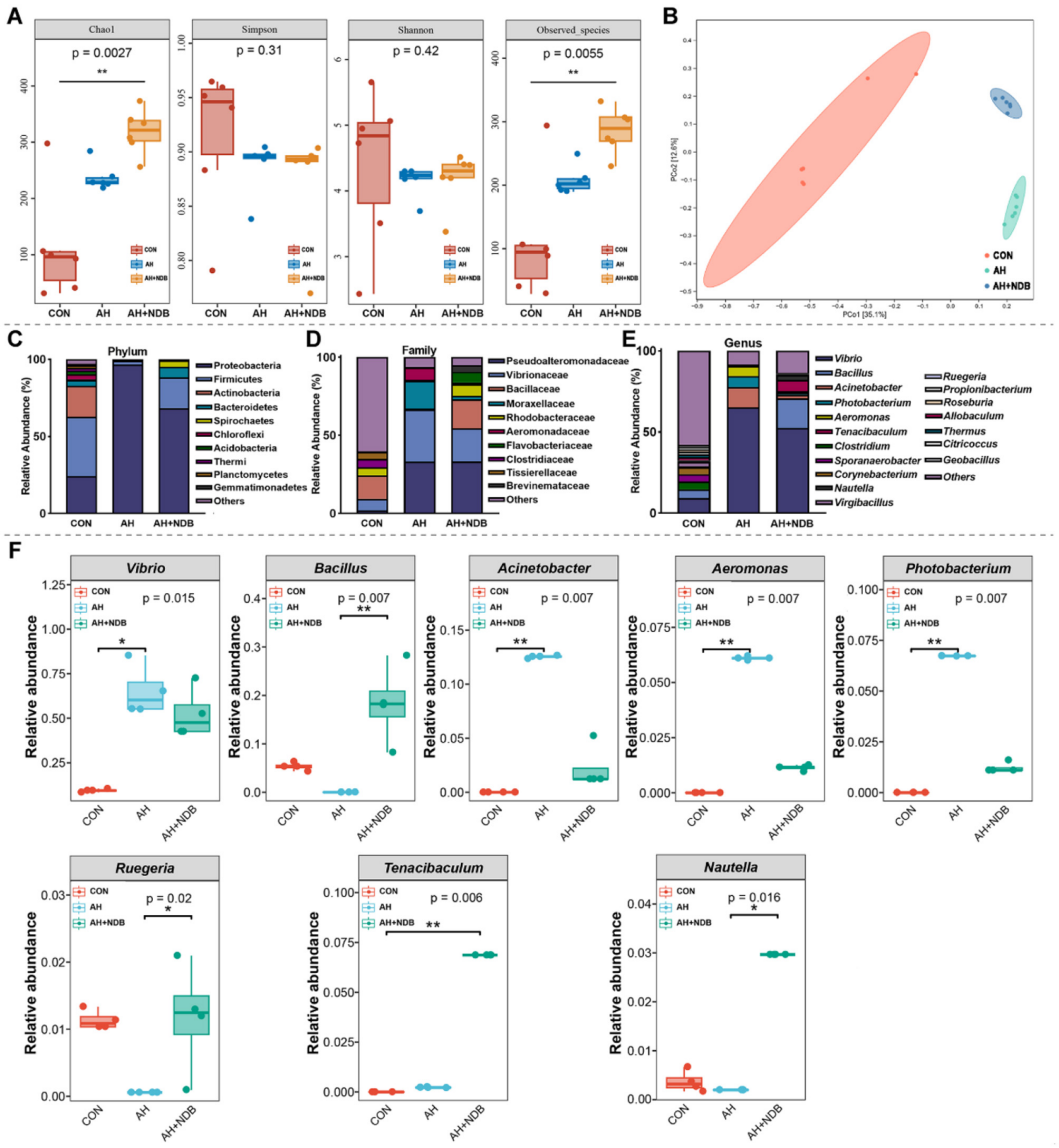
Influence on the modulation of gut microbiota structure in black sea bream. **(A)** Alpha diversity of gut microbiota in black sea bream. **(B)** Principal coordinate analysis (PCoA) of microbial β-diversity. **(C)** Classification at the phylum level. **(D)** Classification at the family level. **(E)** Classification at the genus level. **(F)** The changes of dominant bacteria *Vibrio, Bacillus, Acinetobacter, Aeromonas, Photobacterium, Ruegeria, Tenacibaculum, Nautella* in the intestinal tract. The data are expressed as the mean ± SD (*n* = 3). * *p* < 0.05, **p < 0.01 and ***p < 0.001 by one-way ANOVA and Tukey's *post-hoc* test.

The richness and diversity of microbial communities were evaluated using alpha- and beta-diversity metrics (Figure 6A). Compared with the CON group, the AH+NDB group showed increased species richness, as indicated by higher species (286.17 ± 36.25) and Chao1 (318.83 ± 39.95) indices. However, A reduction in both Shannon (4.18 ± 0.41) and Simpson (0.87 ± 0.05) diversity indices was observed in the AH+NDB group, indicating that dietary supplementation with *B. velezensis* NDB increased microbial richness but paradoxically reduced overall community diversity. Moreover, β-diversity was evaluated through PCoA using weighted UniFrac distance matrices to visualize microbial community structural variation (Figure 6B). Compared with the CON group, the microbial community structure in the AH+NDB group differed markedly from that in the AH group, reflecting that both *B. velezensis* NDB supplementation and *A. hydrophila* infection induced distinct restructuring of the intestinal microbiota in black sea bream.

#### 3.5.2 Composition of gut microbiota

The microbial community structure analysis showed that at the phylum level, *A. hydrophila* infection in the AH group increased the relative abundance of Proteobacteria (96.6%), while decreased the relative abundances of Firmicutes (2.5%), Actinobacteria (0.4%) and Bacteroidetes (0.3%) as compared to the CON group (Figure 6C). Compared with the CON group, dietary supplementation with *B. velezensi*s NDB decreased the relative abundances of Proteobacteria (68.4%) and Actinobacteria (0.1%), and increased the relative abundances of Firmicutes (19.9%), Bacteroidetes (7.0%) and Spirochaetes (4.1%). At the family level, the relative abundances of Vibrionaceae, Moraxellaceae and Aeromonadaceae markedly increased in the AH group (33.38%, 17.82%, and 7.73%, respectively) as compared to the CON group (7.48%, 0.06% and 0%, respectively) (Figure 6D). However, dietary supplementation with *B. velezensi*s NDB in the AH+NDB group decreased the relative abundances of Vibrionaceae (21.31%), Moraxellaceae (2.26%), and Aeromonadaceae (1.06%) as compared to the AH group. Compared with the CON group, the relative abundances of Bacillaceae and Rhodobacteraceae decreased in the AH group from 14.91% and 5.10% to 0.66% and 0.70%, respectively, whereas increased to 18.51% and 7.49% in the AH+NDB group. At the genus level, *A. hydrophila* infection in the AH group significantly increased the relative abundance of *Vibrio* (65.19%), *Acinetobacter* (12.53%), and *Aeromonas* (6.1%) (*p* < 0.01), while decreased the relative abundance of *Bacillus, Ruegeria*, and *Sporanaerobacter* as compared to the CON group (Figures 6E,F). Compared with the AH group, dietary supplementation with *B. velezensis* NDB in the AH+NDB group markedly decreased the relative abundances of *Vibrio* (52.53%), *Acinetobacter* (2.22%), and *Aeromonas* (1.13%) (*p* < 0.01), and increased the relative abundances of *Bacillus* (18.24%), *Ruegeria* (1.18%), and *Tenacibaculum* (6.86%) (*p* < 0.05).

#### 3.5.3 Analysis of species differences in gut microbiota

**Figure 7:**
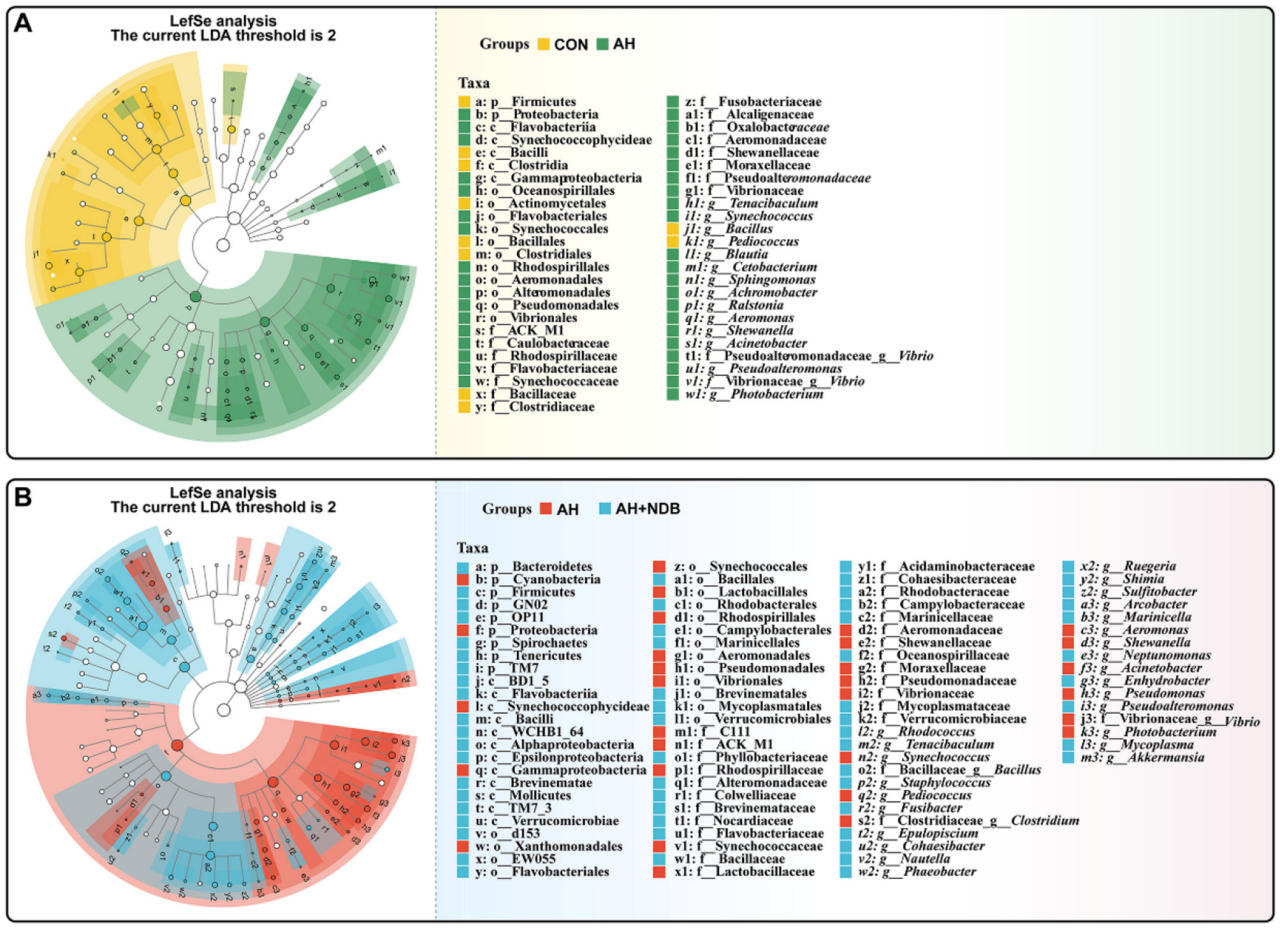
Linear discriminant analysis effect size (LEfSe) to analyse differences in microbial community abundance. The findings with regards to phylum and genus are shown in the plot Linear Discriminant Analysis (LDA) score > 2 (*p* < 0.05). **(A)** LEfSe analysis of relative abundance of gut microbial populations in the AH group *vs*. the CON group. The green bars show the higher relative abundance of these bacterial taxa in the AH group, while the yellow bars show the higher relative abundance of bacterial taxa in the CON group. **(B)** LEfSe analysis of relative abundance of gut microbial populations in the AH+NDB group *vs*. the AH group. Light blue bars represent significantly higher relative abundance of these bacterial taxa in the AH+NDB group, while red bars represent significantly higher relative abundance of these bacterial taxa in the AH group.

The LEfSe method with linear discriminant analysis (LDA) score >2 and statistical significance defined by a *p*-value < 0.05 (Kruskal-Wallis test) was employed to identify key prokaryotic taxa with significantly altered abundance in the gut microbiota of black sea bream (Figure 7). Compared with the CON group, *A. hydrophila* infection in the AH group significantly increased relative abundance of several genus taxa, including *Tenacibaculum, Synechococcus, Blautia, Cetobacterium, Sphingomonas, Achromobacter, Ralstonia, Aeromonas, Shewanella, Acinetobacter, Pseudoalteromonas, Vibrio*, and *Photobacterium* (Figure 7A). Compared with the AH group, dietary supplementation with *B. velezensis* NDB in the AH+NDB group markedly increased relative abundance of some genus taxa, including *Rhodococcus, Tenacibaculum, Bacillus, Staphylococcus, Fusibacter, Epulopiscium, Cohaesibacter, Nautella, Phaeobacter, Ruegeria, Shimia, Sulfitobacter, Arcobacter, Marinicella, Neptunomonas, Enhydrobacter, Pseudoalteromonas, Mycoplasma*, and *Akkermansia* (Figure 7B).

#### 3.5.4 Functional prediction of gut microbiota

**Figure 8:**
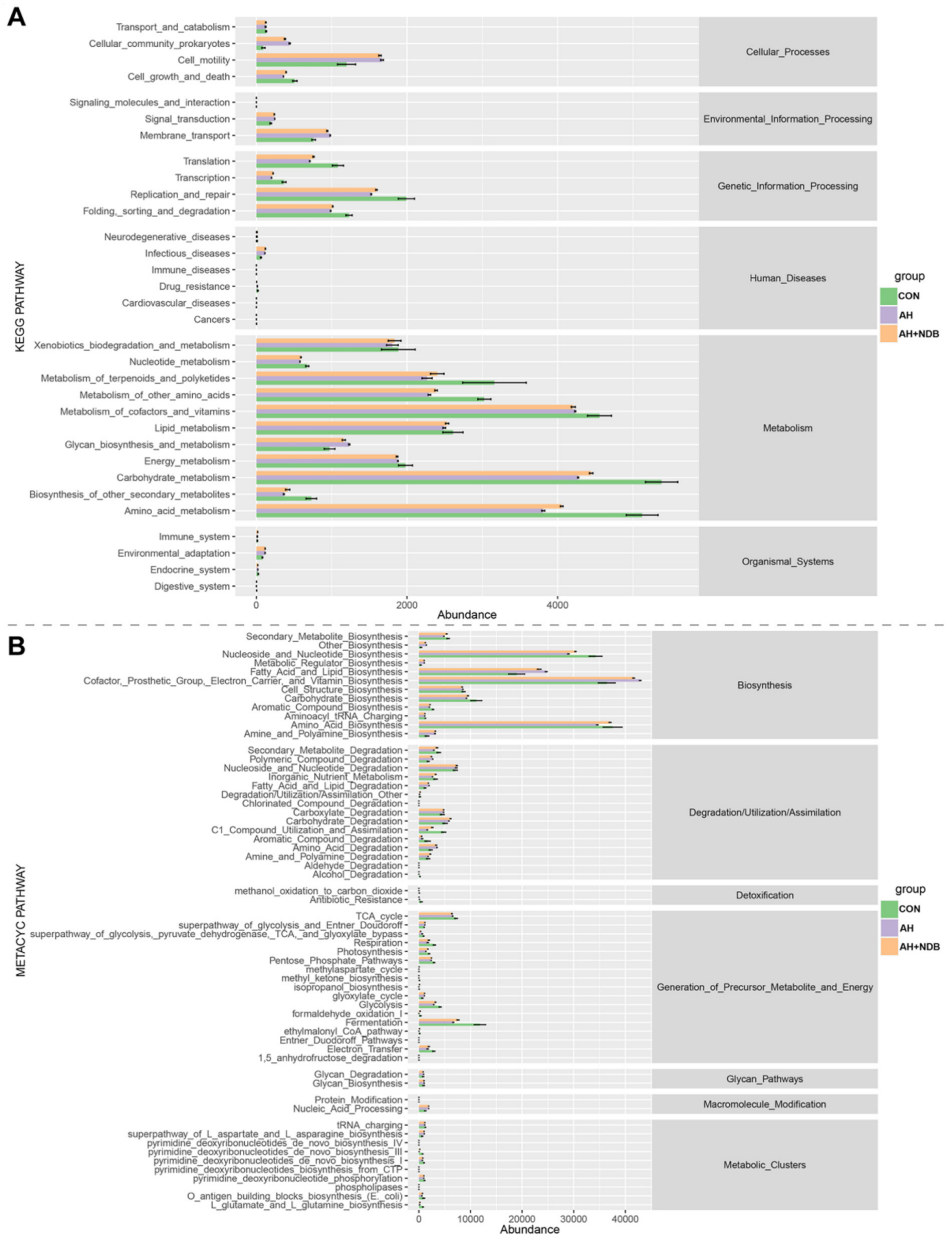
Statistically significant correlations between genus-level gut microbiota and key metabolic pathways visualized through a chord diagram. **(A)** Statistical analysis of KEGG differential metabolic pathways. **(B)** Statistical analysis of MetaCyc differential metabolic pathways.

In this study, functional prediction of the gut microbiota for KEGG and MetaCyc pathways was performed using the PICRUSt2 software. As shown in Figure 8A, the microbial functions of gut microbiota were mainly enriched in KEGG pathways encompassing six major categories: cellular processes, environmental information processing, genetic information processing, human diseases, metabolism, and organismal systems. Compared with the CON group, the AH and AH + NDB groups increased some metabolic pathways, including cellular community prokaryotes, cell motility, signal transduction, membrane transport, infectious diseases. Although most pathways exhibited reduced abundance in both the AH and AH + NDB groups relative to the CON group, supplementation with *B. velezensis* NDB (AH + NDB group) specifically enhanced some pathways such as replication and repair, metabolism of terpenoids and polyketides, metabolism of other amino acids, lipid metabolism, carbohydrate metabolism and amino acid metabolism as compared to the AH group. As illustrated in Figure 8B, the functional profiles of gut microbiota were predominantly enriched in MetaCyc pathways containing seven major categories: biosynthesis, degradation/utilization/assimilation, detoxification, generation of precursor metabolite and energy, glycan pathways, macromolecule modification, and metabolic clusters. Notably, *A. hydrophila* infection in the AH group increased the pathways related to fatty acid and lipid biosynthesis as well as cofactor, prosthetic group, electron, carrier and vitamin biosynthesis as compared to the CON and AH + NDB groups. Moreover, while most pathways exhibited reduced abundance in both AH and AH+NDB groups compared to the CON group, supplementation with *B. velezensis* NDB (AH + NDB group) increased some pathways such as secondary metabolite biosynthesis, nucleoside and nucleotide biosynthesis, carbohydrate biosynthesis, amino acid biosynthesis, glycolysis, and fermentation relative to the AH group.

#### 3.5.5 Analysis of the correlation between gut microbiota and metabolic pathways

**Figure 9:**
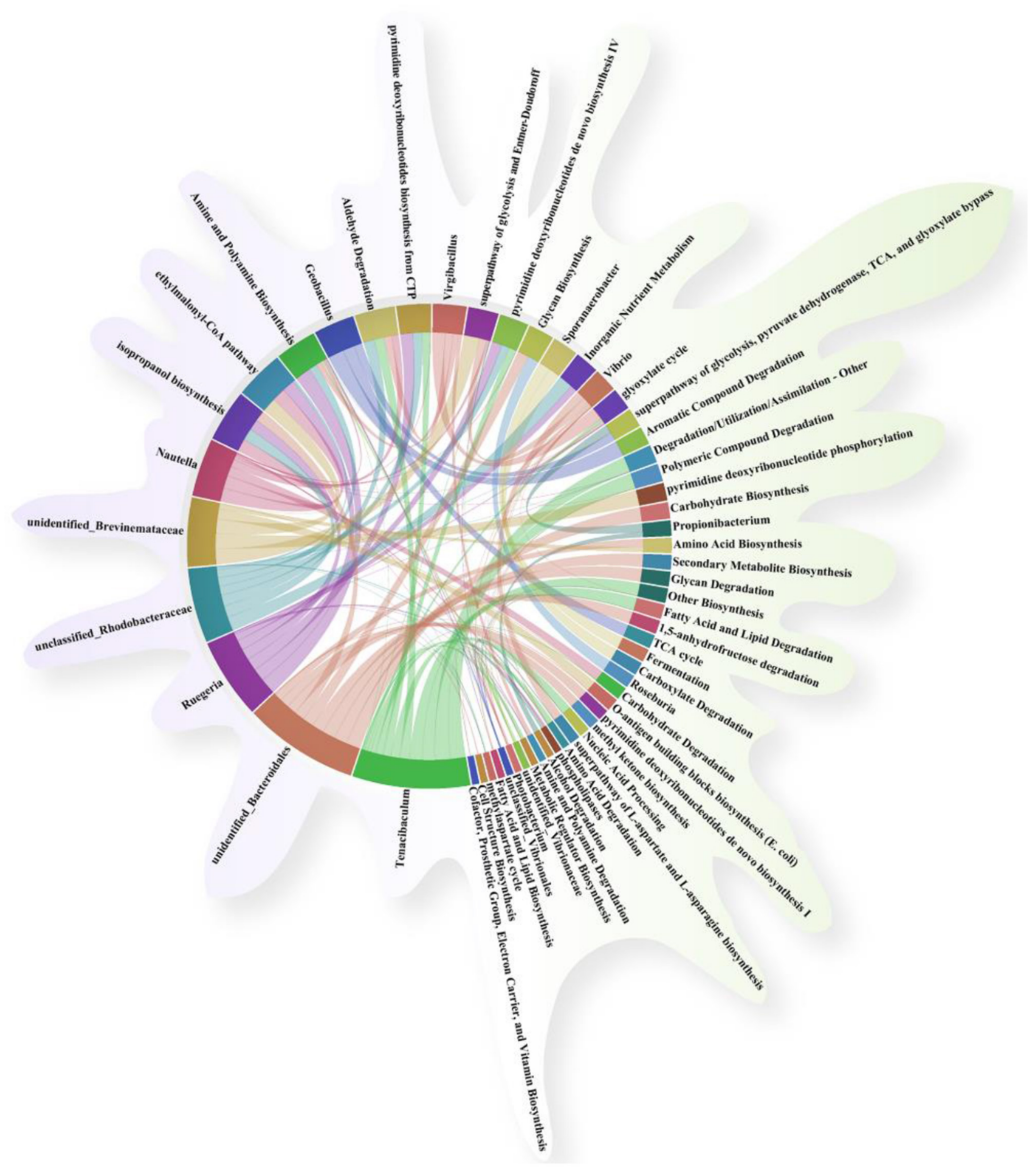
Association and chord diagram between gut microbiota and key metabolic pathways (Spearman's correlation analysis, *p* < 0.05). The thickness of the lines represents the strength of the association, and different colors distinguish between microbial groups and metabolic pathway categories.

Correlation analysis was performed to investigate the relationship between the gut microbiota of genus-level microorganisms and key metabolic pathways. As shown in Figure 9, fatty acid and lipid degradation pathways are mainly driven by *Vibrio*, and the two are significantly positively correlated, suggesting that this genus plays a dominant role in lipid metabolism. TCA cycle is strongly positively correlated with *Geobacillus*. The L-aspartic acid and L-asparagine biosynthesis superpathway is mainly associated with the following bacterial genera: *Photobacterium, Aeromonas, Vibrionaceae, Acinetobacter, Vibrionales*, and *Vibrio* suggesting that these groups are jointly involved in the amino acid synthesis process. Acid Biosynthesis involves multiple taxonomic groups, including *Acinetobacter, Aeromonas, Vibrionaceae, Photobacterium, Vibrio, Tenacibaculum, Bacillus, Brevinemataceae*.

#### 3.5.6 Gut microbiota and inflammation index correlation analysis

**Figure 10:**
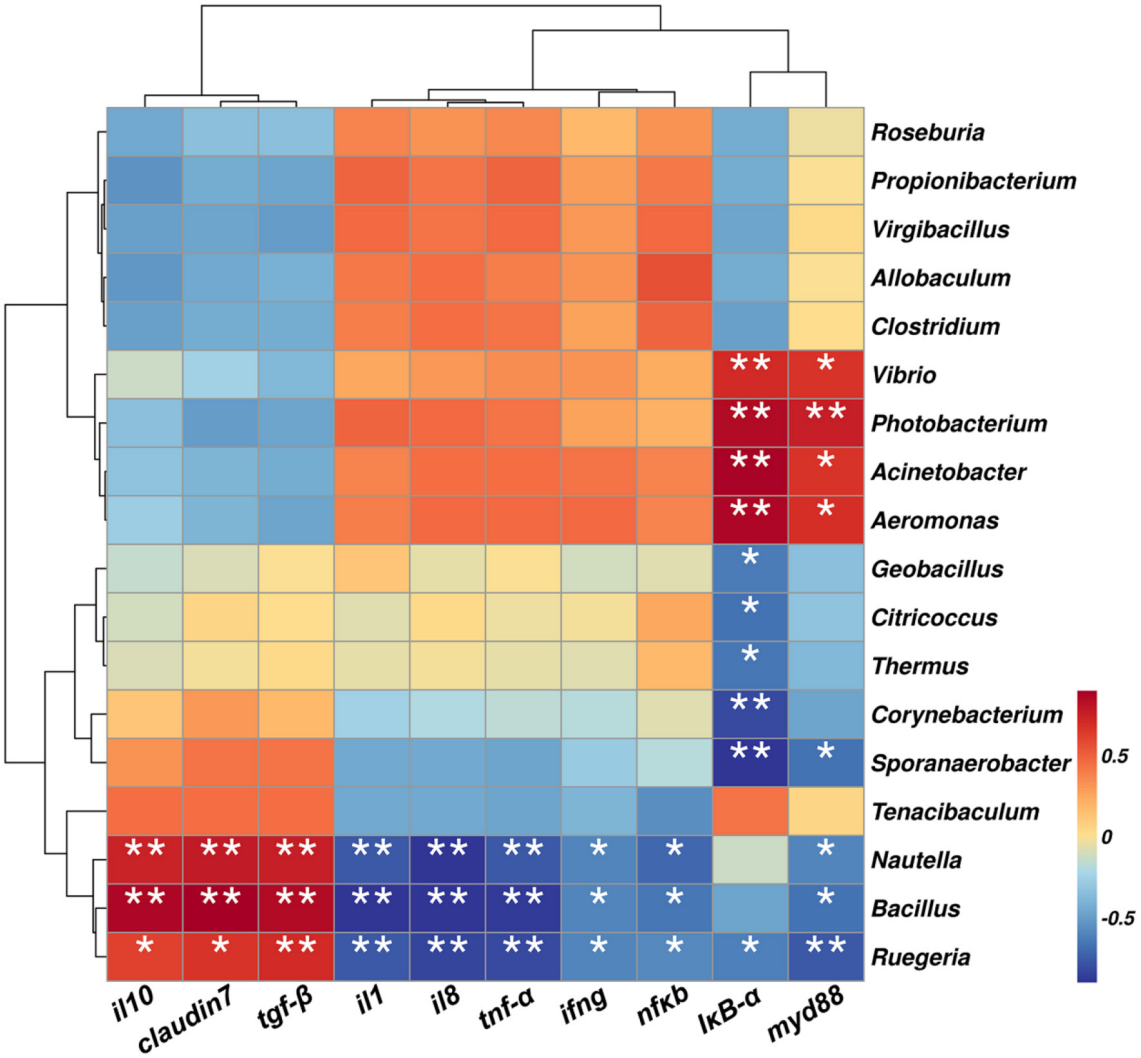
Correlation heatmap between key inflammatory markers and gut microbial genera. Positive and negative correlations are color-coded. * *p* < 0.05, **p < 0.01 and ***p < 0.001 by one-way ANOVA and Tukey's *post-hoc* test.

In this experiment, the 18 top most abundant bacterial genera were selected for correlation analysis with key inflammatory markers. As shown in Figure 10, *Ruegeria, Bacillus*, and *Nautella* were significantly positively correlated with the inhibition of the inflammatory cytokines (*il10* and *tgf-*β) and the tight junction gene *claudin7* (*p* < 0.01), and significant negative correlations with pro-inflammatory markers *nfkb, ifng, il8, il1, I*κ*B-*α and *tnf-*α (*p* < 0.05). In contrast, *Vibrio, Photobacterium, Acinetobacter*, and *Aeromonas* showed opposite trends, being negatively correlated with the anti-inflammatory markers (*il10* and *tgf-*β) and the tight junction gene *claudin7* and positively correlated with pro-inflammatory factors, particularly *I*κ*B-*α and *myd88* (*p* < 0.05).

## 4 Discussion

Enteritis caused by *A. hydrophila* is a major health concern in marine aquaculture, often leading to disrupted intestinal morphology, inflammation, oxidative stress, and gut microbial dysbiosis in fish species (Ma et al., 2022; Xu et al., 2022). Marine environments are a rich reservoir of probiotics, and numerous studies have demonstrated that marine-derived probiotics produce novel bioactive compounds with anticancer, antibacterial, immunomodulatory, antioxidant, anti-inflammatory, and antiviral properties (Eze et al., 2023). In the present study, dietary supplementation with marine-derived *B. velezensis* NDB significantly alleviated *A. hydrophila*-induced enteritis in black sea bream by enhancing mucosal immunity, reducing oxidative damage, and modulating the gut microbiota. These findings underscore the potential of *B. velezensis* NDB as a functional probiotic in marine aquaculture. *A. hydrophila* infection induced typical enteric symptoms, including gill and abdominal hemorrhages, impaired intestinal architecture, decreased goblet cell abundance, and severe inflammatory infiltration (Figures 2, 3), consistent with previous descriptions of bacterial enteritis in fish (Nassar et al., 2024). Supplementation with *B. velezensis* NDB at 1.43 × 10^8^ CFU/g significantly attenuated these pathological changes (Rahman et al., 2023), reduced mortality, and promoted growth performance (Table 2) (Aini et al., 2024). Notably, the treatment preserved villus morphology, increased goblet cell numbers, and reduced inflammatory cell infiltration (Liao et al., 2023). Goblet cells are essential for maintaining mucosal defense, as they secrete protective mucus and facilitate pathogen clearance (Kuebutornye et al., 2020). The restoration of epithelial integrity highlights the capacity of *B. velezensis* NDB to maintain the physical barrier function of the intestine under pathogen-induced stress.

Oxidative stress is a hallmark of bacterial infections and contributes to tissue damage. Measurement of hepatic antioxidant enzyme activities can indirectly assess the impact of intestinal inflammation on systemic oxidative homeostasis (Goulart et al., 2020). Notably, SOD and CAT indicate hepatic antioxidant capacity, while MDA reflects oxidative damage. Pepsin activity in the stomach and intestine reflects protein digestion capacity (Zuo et al., 2024). SOD and CAT are considered molecular biomarkers for assessing the oxidative stress in aquatic organisms (Kembou-Ringert et al., 2023; Qian et al., 2024). MDA is a byproduct of the interaction between free radicals and lipids, and its concentration can indirectly reflect levels of lipid peroxidation and free radical generation, indicating the extent of free radical damage to cellular structures (Suharso et al., 2022). In this study, following infection with *A. hydrophila*, CAT and SOD activities decreased in black sea bream (Figure 4), consistent with previous reports (Deng et al., 2023). *B. velezensis* NDB supplementation restored SOD and CAT activity and decreased MDA levels, suggesting that this strain could enhance endogenous antioxidant capacity and protects liver function.

Immune markers are effective indicators for assessing the health status of fish (López Nadal et al., 2020). In intestinal inflammation, *tgf-*β promotes intestinal mucosal repair by regulating immune cell proliferation and differentiation (Peng et al., 2022). *Il1* and *il8*, as key pro-inflammatory factors, mediate inflammatory cascades and neutrophil chemotaxis, respectively (Zhang et al., 2020; Leong et al., 2024). The activation of the NF-κB pathway is the core mechanism triggering the inflammatory storm (Yu et al., 2020), it induces pro-inflammatory gene expression (e.g., *il1, tnf-*α) via the *myd88*-dependent pathway, accompanied by the phosphorylation and degradation of (Reis et al., 2016). In this study, *A. hydrophila* infection significantly upregulated NF-κB pathway-related genes (*myd88, nf*κ*b*) and downstream pro-inflammatory factors (*il1, tnf-*α, *ifng*) (Figure 5). This confirms the excessive inflammatory response triggered by the pathogen. Claudin family proteins are key molecules in maintaining intestinal barrier function (Wang K. et al., 2024; Shen and Mu, 2024). Among them, *claudin 7* not only participates in the formation of tight junctions between cells but also influences intestinal mucosal homeostasis by regulating the expression of cell-matrix adhesion molecules (Ding et al., 2012). In this study, pathogen infection led to a significant decrease in *claudin 7* expression (Figure 5J), which was significantly negatively correlated with increased intestinal permeability and tissue pathological damage (Figure 3), suggesting that impaired barrier function is an important trigger for inflammatory spread. Notably, *B. velezensis* NDB intervention not only significantly downregulated pro-inflammatory factors (*il1, tnf-*α) but also restored *claudin 7* expression (reconstructing the intestinal physical barrier), an effect synergistically enhanced by the upregulation of anti-inflammatory factors *il10* and *tgf-*β (Figures 5H, I), collectively promoting the restoration of immune homeostasis. This aligns with findings in Nile tilapia (*Oreochromis niloticus*) (Zhao et al., 2023), suggesting that probiotics can modulate immune pathways to alleviate intestinal inflammation.

The gastrointestinal tract is the primary colonization site for probiotics (Goh et al., 2021). By colonizing the intestinal mucosal surface, probiotics competitively occupy adhesion sites that would otherwise be utilized by pathogenic bacteria, thereby inhibiting pathogenic colonization (Yousuf et al., 2023). Additionally, enhanced adhesion to intestinal epithelial cells amplifies the probiotics' potential to stimulate the immune system, thereby protecting intestinal epithelial cells from mechanical damage (Govindaraj et al., 2021). In this research, analysis of gut microbiota indicated that after four weeks of feeding with *B. velezensis* NDB in the diet, the bacterium was found to colonize in the intestine, with the highest abundance in AH+NDB group (Figure 6). Following *A. hydrophila* infection, *Aeromonas* had the highest abundance in the AH group but decreased in the AH+NDB group, suggesting that *B. velezensis* NDB has an inhibitory effect on *A. hydrophila* infection. Proteobacteria, Firmicutes, Actinobacteria, and Bacteroidetes were dominant in the intestinal flora, similar to previous research (Lin et al., 2024). Following infection with *A. hydrophila*, the abundance of Proteobacteria increased significantly, which could be regarded as a microbial indicator of intestinal flora imbalance. Many important pathogens belong to the Gammatimonadetes, including *Salmonella* (enteritis and typhoid), *Yersinia* (plague), *Vibrio* (cholera), and *Pseudomonas aeruginosa* (pulmonary infections or cystic fibrosis-related infections) (Idola et al., 2023). Marine bacteria in the Vibrionaceae family play significant roles in the marine geochemical cycle and function as symbionts and opportunistic pathogens of aquatic animals and humans (Kumar et al., 2023). Aeromonadaceae is a family commonly found in seawater, freshwater, silt, sewage and feces, while several species of this family are pathogenic to fish, amphibians and humans (Lopez-Zavala et al., 2018). The family Moraxellaceae contains a variety of opportunistic pathogens such as *Acinetobacter baumannii* and *Moraxella catarrhalis* (Knoot et al., 2023). This finding indicated that dietary supplementation of *B. velezensis* NDB reversed this imbalance, promoting the growth of beneficial taxa and suppressing potential pathogens.

In addition, functional prediction of the gut microbiota and their metabolic pathways was performed to further investigate the potential mechanism whereby dietary supplementation of *B. velezensis* NDB alleviated *A. hydrophila*-induced enteritis in black sea bream. This study reveals four potential synergistic mechanisms: First, competitive colonization and pathogenic bacteria inhibition: *B. velezensis* NDB significantly reduced the abundance of pathogenic bacteria belonging to the genera *Aeromonas* and *Vibrio* genera (Figure 7), consistent with its ability to construct a biochemical protective barrier through secondary metabolites (Sommerfeld et al., 2024). Previous studies indicated that terpenoids could inhibit the NF-κB signaling pathway by downregulating related genes and enriching pathways such as terpenoid/polyketide metabolism and carbohydrate metabolism, directly disrupting the cell membranes of *A. hydrophila* and inhibiting its reproduction (Salminen et al., 2008; Sun et al., 2023). Also, this study showed downregulated pro-inflammatory genes (*nf*κ*b, tnf-*α; Figure 5), and highly abundant KEGG pathways (e.g., terpenoids/polyketides metabolism, carbohydrate biosynthesis, amino acid biosynthesis pathways and nucleoside and nucleotide biosynthesis; Figure 8), suggesting their dual roles in inhibiting pathogen proliferation and inflammatory activation. Secondly, dietary supplementation with *B. velezensis* NDB can regulate metabolic pathways by significantly enhancing multiple KEGG pathways, including cofactor/vitamin metabolism, carbohydrate biosynthesis, and amino acid biosynthesis (Figure 8). These pathways not only participate in the synthesis of antimicrobial substances but also provide energy through efficient glycolysis/tricarboxylic acid cycle. (1) Energy provision: they support antioxidant reactions (consistent with the results of increased SOD and CAT activity in (Figure 4); (2) Anti-inflammatory action: they compete for carbon sources to suppress pro-inflammatory molecules (e.g., lipopolysaccharides, LPS) (Choi et al., 2021). Thirdly, dietary supplementation with *B. velezensis* NDB can play a crucial role in the intestinal barrier repair effect. As illustrated in Figures 8, 9, the AH+NDB group activated specific biosynthesis pathways (e.g., fatty acid/lipid biosynthesis and cofactor/prosthetic group/electron carrier/vitamin biosynthesis), thereby supplying energy, cofactors, and lipid precursors to sustain intestinal epithelial cell function and directly support the recovery of intestinal physical barrier function (Chen et al., 2023). Fourthly, marine adaptation of *B. velezensis* NDB and its promotion of microbiota remodeling. Originating from the high-salinity environment of Xiangshan Port, *B. velezensis* NDB modulated the intestinal microbiota by significantly enriching the beneficial genera (e.g., *Nautella, Bacillus*, and *Ruegeria*), while continuously inhibiting opportunistic pathogenic genera (e.g., *Aeromonas* and *Vibrio*) (Figure 10). This remodeling contributed to the formation of a healthy microbial community structure (Dawood et al., 2020; Wang et al., 2023b). Collectively, dietary supplementation with *B. velezensis* NDB can protect black sea bream against *A. hydrophila*-induced enteritis via a synergistic mechanism associated with barrier repair, immune regulation, colonization competition, metabolic regulation, and microbiota reshaping.

In addition, this study is subject to several limitations that warrant consideration. Firstly, current dietary supplementation with dietary *B. velezensis* NDB supplementation is limited by the 4-week experimental period, which fails to capture potential long-term effects, necessitating prolonged intervention trials to show that dietary *B. velezensis* NDB can effectively improve the resistance of black sea bream to the pathogenic bacteria *A. hydophila*. Secondly, due to equipment limitations, this study was unable to monitor the behavior of black sea bream over a long period, including at night, which would have provided more comprehensive data to better assess growth performance. Thirdly, current study on dietary supplementation with *B. velezensis* NDB have not yet characterized its effects on nutritional composition parameters of black sea bream muscle tissue, including protein contents, amino acid profiles, and fatty acid composition. Finally, although the present study investigated the alterations of gut microbiota in black sea bream across the CON, AH, and AH+NDB groups, environmental microbiota in aquaculture systems and their potential correlations with intestinal microbiota were not assessed. Collectively, future research should further explore the effects of longer-term feeding *B. velezensis* NDB in black sea bream, particularly those involving promoted growth performance and enhanced muscle nutrients of black sea bream.

## 5 Conclusion

This study demonstrates that dietary supplementation with *B. velezensis* NDB confers significant protective effects against *A. hydrophila*-induced enteritis in black sea bream. The probiotic promoted growth performance, enhanced antioxidant enzyme activities, preserved intestinal morphology, and modulated both pro—and anti-inflammatory immune responses. Additionally, it reshaped the gut microbiota by reducing the abundance of pathogenic bacteria and promoting beneficial taxa, contributing to microbial and immunological homeostasis. Functional predictions suggest that its protective mechanisms involve carbohydrate biosynthesis, amino acid biosynthesis pathways and nucleoside and nucleotide biosynthesis, supporting both antimicrobial activity and host adaptation. Collectively, these findings establish *B. velezensis* NDB as a promising probiotic candidate for enhancing intestinal health and disease resistance in marine aquaculture systems.

## Data Availability

The datasets presented in this study are publicly available. This data can be found here: https://www.ncbi.nlm.nih.gov/sra, accession number: PRJNA1332308.
